# Correction: Stall force measurement of the kinesin-3 motor KIF1A using a programmable DNA origami nanospring

**DOI:** 10.7554/eLife.113474

**Published:** 2026-10-21

**Authors:** Nobumichi Takamatsu, Hiroko Furumoto, Takayuki Ariga, Mitsuhiro Iwaki, Kumiko Hayashi

**Keywords:** Human

Takamatsu N, Furumoto H, Ariga T, Iwaki M, Hayashi K. 2026. Stall force measurement of the kinesin-3 motor KIF1A using a programmable DNA origami nanospring. *eLife* **14**:RP108477. doi: 10.7554/eLife.108477.

Published 25 March 2026

After publication, we became aware of three errors in the Materials and methods section: one in the description of the buffer composition and two in the description of the analytical methods. These errors arose from oversights during manuscript preparation. Because the experiments and analyses were conducted correctly, these corrections do not affect the figures, data, statistical analyses, results, interpretation, or conclusions of the article.

1. Materials and methods, “Kinesin-NS conjugation”:

The SRP90 buffer composition was incomplete, with 10% glycerol inadvertently omitted from the manuscript.

Corrected text:

“SRP90 buffer (90 mM HEPES, 50 mM CH_3_COOK, 2 mM Mg(CH_3_COO)_2_, 1 mM EGTA, 10% glycerol, pH 7.6, adjusted with KOH)”

Original text:

“SRP90 buffer (90 mM HEPES, 50 mM CH_3_COOK, 2 mM Mg(CH_3_COO)_2_, 1 mM EGTA, pH 7.6, adjusted with KOH)”

2. Materials and methods, “Angular fluctuation”:

The number of frames used to calculate the mean direction vector ($\overset{\rightarrow }{R_{t}}$) were incorrectly described.

Corrected text:

$$\displaystyle  \overset{\rightarrow }{R_{t}}=\frac{1}{11}\sum _{j=t-5}^{t+5}\overset{\rightarrow }{v_{j}}.$$

Original text:

$$\displaystyle  \overset{\rightarrow }{R_{t}}=\frac{1}{\omega }\sum _{j=t-\omega /2}^{t+\omega /2+1}\overset{\rightarrow }{v_{t}}.$$

3. Materials and methods, “Rate of relative increase in NS’s length”:

The equation describing the 10-frame moving average of the NS extension ($\overline{L}\left (\tau \right)$) was incorrectly written.

Corrected text:

“$\overline{L}\left (\tau \right)\left (=\left (\sum _{t=\tau -5}^{\tau +4}L\left (t\right)\right)\big/10\right)$ where τ represents a video frame”

Original text:

“$\overline{L}\left (\tau \right)\left (=\left (\sum _{t=10\left (\tau -1\right)+1}^{10\tau}L\left (t\right)\right)\big/10\right)$ where t represents a video frame”

The article has been corrected accordingly.

