# Supplementary material for: Stall force measurement of the kinesin-3 motor KIF1A using a programmable DNA origami nanospring
Source: eLife. 2026 Mar 25;14:RP108477. doi: 10.7554/eLife.108477 (PMC13016605; doi:10.7554/eLife.108477)
Supplement: Supplementary file 1. — Listed below are the plasmids used in this study. [file elife-108477-supp1.docx]

| Plasmid | Description | Backbone |
| --- | --- | --- |
| pSN643 | KIF1A(1-393)::LZ::mScarlet-I::Strep-tagⅡ | pET28a |
| pSN672 | KIF1A(1-393)::LZ::His-tag | pET21a |
| pHF001 | KIF1A(1-393)::LZ::SNAP::His-tag | pET21a |
| pHF002 | KIF1A(1-393)(P305L)::LZ:: SNAP::His-tag | pET21a |
| pHF003 | KIF1A(1-393)(V8M)::LZ:: SNAP::His-tag | pET21a |
| pHF004 | KIF1A(1-393)(A255V)::LZ:: SNAP::His-tag | pET21a |
| pHF005 | KIF1A(1-393)::LZ::SNAP:: Strep-tagⅡ | pET28a |
| pYS05 | KIF5B(1-560)(G234A)::RA::SNAP::His-tag | pET17b |

RA: RA linker
