# Supplementary material for: Stall force measurement of the kinesin-3 motor KIF1A using a programmable DNA origami nanospring
Source: eLife. 2026 Mar 25;14:RP108477. doi: 10.7554/eLife.108477 (PMC13016605; doi:10.7554/eLife.108477)
Supplement: Supplementary file 2. — In the sequences of staples handle 32A and 32B, italicized regions indicate single-stranded DNA (ssDNA) handle sequences. [file elife-108477-supp2.docx]

| **Name** | **Sequence (5’ to 3’)** |
| --- | --- |
| handle 32A staple | AATCGGAACCCTAAAGGAAAAACCGTCTATCA  *CCCACCTATTTTCACCCCACCCTTCCCCCAAC* |
| handle 32B staple | *CCTTATCCCAAACCCCTCCCCCAACCATTCCC*  TTACCAGTATAAATGAGTAATGTGTAGGTAAA |
| oligo 32A* | NH_2_/GTTGGGGGAAGGGTGGGGTGAAAATAGGTGGG |
| oligo 32B* | GGGAATGGTTGGGGGAGGGGTTTGGGATAAGG/NH_2_ |
