# Supplementary material for: Stall force measurement of the kinesin-3 motor KIF1A using a programmable DNA origami nanospring
Source: eLife. 2026 Mar 25;14:RP108477. doi: 10.7554/eLife.108477 (PMC13016605; doi:10.7554/eLife.108477)
Supplement: Supplementary file 3. — Sequences in italics indicate the handle site. [file elife-108477-supp3.docx]

| **Sequence (5’ to 3’)** | **Name** |
| --- | --- |
| ATAAGTGCCGTGGGGAAAGCTATTAAAGAACGTGGAC | Core Staple_01 |
| ATTAGCGGGGTAAGGGAAGGTTGAGTGTTGTTCCAGT | Core Staple_02 |
| AGGCTGAGACTGGGCGCTGTCCCTTATAAATCAAAAG | Core Staple_03 |
| CCTATTATTCTGCGTAACCATCCTGTTTGATGGTGGT | Core Staple_04 |
| AACAGTTAATGCGCCGCTAAGCGGTCCACGCTGGTTT | Core Staple_05 |
| GTCAGTGCCTTTTGACGAGCCCTTCACCGCCTGGCCC | Core Staple_06 |
| AGGAGTGTACTTTAGAATCTCTTTTCACCAGTGAGAC | Core Staple_07 |
| GTAAGCGTCATCCGATTAAAGGCGGTTTGCGTATTGG | Core Staple_08 |
| GAAAGCGCAGTACGCCAGAAGCTGCATTAATGAATCG | Core Staple_09 |
| AACAAATAAATTCAGTGAGCTGCCCGCTTTCCAGTCG | Core Staple_10 |
| CAGGTCAGACGCCATCACGAGTGAGCTAACTCACATT | Core Staple_11 |
| GCCGCCGCCAGCTTCTTTGGAAGCATAAAGTGTAAAG | Core Staple_12 |
| CTCAGAGCCGCTGAGTAGATATCCGCTCACAATTCCA | Core Staple_13 |
| CTCAGAACCGCCTGGTAATATCATGGTCATAGCTGTT | Core Staple_14 |
| ACCGGAACCGCGCCATTGCTTGAGGATCCCCGGGTAC | Core Staple_15 |
| ATAATCAAAATATACCTACTGCCTGTTCTTCGCGTCC | Core Staple_16 |
| AGCCCCCTTATAATGGATTTCACGGTCATACCGGGGG | Core Staple_17 |
| TAGCGCGTTTTGTCACACGTGTGGTGCTGCGGCCAGA | Core Staple_18 |
| AGAATCAAGTTTGGCCAACATCCAGCGCAGTGTCACT | Core Staple_19 |
| ATCGATAGCAGGAAAGCGTCAGCCAGCGGTGCCGGTG | Core Staple_20 |
| AGCAAGGCCGGATTTTTGAAAATCGTTAACGGCATCA | Core Staple_21 |
| AGCAAAATCACCGAACTGACGTCATAAACATCCCTTA | Core Staple_22 |
| ACCGACTTGAGAAAATACCCCTGCGGCTGGTAATGGG | Core Staple_23 |
| ATTATTCATTAAAAACAGAGGCGCGGTTGCGGTATGA | Core Staple_24 |
| TTGAGGGAGGGCCGCCTGCGGTCATTGCAGGCGCTTT | Core Staple_25 |
| GCCAAAGACAAGCAGCAAACCAGCTTACGGCTGGAGG | Core Staple_26 |
| ATCAATAGAAATGCTGAACACGGCAGCACCGTCGGTG | Core Staple_27 |
| GACACCACGGAAATATCTGGCTGGTCTGGTCAGCAGC | Core Staple_28 |
| AAAGGTGGCAAGAAAGGAAGGAACGTGCCGGACTTGT | Core Staple_29 |
| TATGTTAGCAATCTTTAGGCTGGCAGCCTCCGGCCAG | Core Staple_30 |
| CTGGCATGATTGAGCCGTCTCCGGCAAACGCGGTCCG | Core Staple_31 |
| AACGCAATAATTTAGAAGTATGAAGGGTAAAGTTAAA | Core Staple_32 |
| CGAACAAAGTTACAACTCGCCCGTAAAAAAAGCCGCA | Core Staple_33 |
| AGCCCTTTTTATTATTAATAGTTGGGCGGTTGTGTAC | Core Staple_34 |
| ACAATGAAATATCATTTTGATCAAACTTAAATTTCTG | Core Staple_35 |
| TTGAGTTAAGCGGAGCGGAAGCTCTCACGGAAAAAGA | Core Staple_36 |
| CGCTAATATCATCAGATGATGTGAGAGATAGACTTTC | Core Staple_37 |
| CACCCTGAACATGATTGTTAACGTACAGCGCCATGTT | Core Staple_38 |
| GAAGCGCATTAGAAGGGTTGGGAACGGATAACCTCAC | Core Staple_39 |
| CCTTTACAGAGTTTGCACGGCCAGTGCCAAGCTTTCA | Core Staple_40 |
| TTTGTTTAACGCGTAGATTAGGGTTTTCCCAGTCACG | Core Staple_41 |
| ATTTATCCCAATATACAGTGATGTGCTGCAAGGCGAT | Core Staple_42 |
| TTTGCCAGTTAGAAACAATGCCTCTTCGCTATTACGC | Core Staple_43 |
| AACGCTAACGAGAATACCAGCTGCGCAACTGTTGGGA | Core Staple_44 |
| CCCAGCTACAAGAATTATTCCGGAAACCAGGCAAAGC | Core Staple_45 |
| CCTTAAATCAAGAAGATGACACTCCAGCCAGCTTTCC | Core Staple_46 |
| GAACCTCCCGAAAATTAATGGGGACGACGACAGTATC | Core Staple_47 |
| CGGTATTCTAAATTACCTTTGGGCGCATCGTAACCGT | Core Staple_48 |
| AGCAAGCAAATATCAATATTTGACCGTAATGGGATAG | Core Staple_49 |
| TTCATCGTAGGCTGTAAATACCCGTCGGATTCTCCGT | Core Staple_50 |
| CTCATCGAGAACTTAGAATAGCTTTCATCAACATTAA | Core Staple_51 |
| CCAAGAACGGGTAGATTAATCAAAAATAATTCGCGTC | Core Staple_52 |
| ATCAATAATCGGAATTTATAAATCAGCTCATTTTTTA | Core Staple_53 |
| CCATCCTAATTTACCTTTTAATATTTTGTTAAAATTC | Core Staple_54 |
| AGATAAGTCCTTATATAACGATTGTATAAGCAAATAT | Core Staple_55 |
| TAATGCAGAACTCCAATCGCGGTTGATAATCAGAAAA | Core Staple_56 |
| CAGACGACGACCTTTTTCAAATCGTAAAACTAGCATG | Core Staple_57 |
| AGTACCGACAACTTCTGACGAGTCTGGAGCAAACAAG | Core Staple_58 |
| CATTTTCGAGCCCGACCGTAGAGATCTACAAAGGCTA | Core Staple_59 |
| AACAACGCCAAAGAATAAAGATAAATTAATGCCGGAG | Core Staple_60 |
| ACAGTAGGGCTTAAAAAGCCTATCACCATCAATATGATA | Core Staple_61 |
| TTTAGAGCTTGACCGAGAGGGCCGCCACCCTCAGAACCG | Core Staple_62 |
| GGCGAGAAAGGTTTGCTCACACCCTCAGAGCCACCAC | Core Staple_63 |
| AGCGGGCGCTACCTCAAGAAGCAAGCCCAATAGGAAC | Core Staple_64 |
| GGTCACGCTGCGAAACATGTGAGTTTCGTCACCAGTA | Core Staple_65 |
| CGCGCTTAATGCCCCCTGCTAGCATTCCACAGACAGC | Core Staple_66 |
| CTATGGTTGCTGAGTAACAACGATCTAAAGTTTTGTC | Core Staple_67 |
| TGCTTTCCTCGGGTAATAAGTAAATGAATTTTCTGTA | Core Staple_68 |
| TAAACAGGAGGACATGGCTAACTTTCAACAGTTTCAG | Core Staple_69 |
| ACAGGAACGGTCTCTGAATAGGAACAACTAAAGGAAT | Core Staple_70 |
| TGTTTTTATAACCTCATTATTCACGTTGAAAATCTCC | Core Staple_71 |
| AAAGAGTCTGTATTGGCCTAAGGAGCCTTTAATTGTA | Core Staple_72 |
| TTGTAGCAATACATTGACACTTTCGAGGTGAATTTCT | Core Staple_73 |
| CATCACTTGCCCACCAGAAGATAGTTGCGCCGACAAT | Core Staple_74 |
| TATCGGCCTTGCACCCTCACACGCATAACCGATATAT | Core Staple_75 |
| TATTACCGCCACTCCCTCAGCAGGGAGTTAAAGGCCG | Core Staple_76 |
| CGCTCATGGAACACCGGAAACCCTCAGCAGCGAAAGA | Core Staple_77 |
| CAATCGTCTGATAGCGTTTGTAGCAACGGCTACAGAG | Core Staple_78 |
| CAGATTCACCACATCGGCAACTTTTTCATGAGGAAGT | Core Staple_79 |
| AAGGGACATTCTGCCTTTAAAATACGTAATGCCACTA | Core Staple_80 |
| CCTTCTGACCTCACCGTAAAAACGAAAGAGGCAAAAG | Core Staple_81 |
| GCACAGACAATAAACGTCACATCTTTGACCCCCAGCG | Core Staple_82 |
| TCTTTAATGCGCAGTAGCAAACAAAGTACAACGGAGA | Core Staple_83 |
| CATCGCCATTACCATTTGGATAAATTGTGTCGAAATC | Core Staple_84 |
| CAGCAGAAGATAAGGTGAATTACTTAGCCGGAACGAG | Core Staple_85 |
| CAGTATTAACAAAGGTAAAATAAGGGAACCGAACTGA | Core Staple_86 |
| GCTGAGAGCCAAAGGGCGAACAGATGAACGGTGTACA | Core Staple_87 |
| AAGCATCACCTATTCATATTGGCTGACCTTCATCAAG | Core Staple_88 |
| AACCCTCAATCATAAGTTTACCGGATATTCATTACCC | Core Staple_89 |
| AATCAACAGTTCATATAAAGCTGCTCATTCAGTGAAT | Core Staple_90 |
| TATCTAAAATAACGTAGAAAGAAACACCAGAACGAGT | Core Staple_91 |
| CTAATAGATTAAAGACTCCGATGGTTTAATTTCAACT | Core Staple_92 |
| CATTTGAGGATAACGGAATACCTTATGCGATTTTAAG | Core Staple_93 |
| CAAACAATTCGACCAGAAGCAGTCAGGACGTTGGGAA | Core Staple_94 |
| TTGCCCGAACGAGAAAAGTTAAAACGAACTAACGGAA | Core Staple_95 |
| GAGTAACATTAGCAATAGCAGAAAGATTCATCAGTTG | Core Staple_96 |
| AACCACCAGAACCAATAATCATTCAACTAATGCAGAT | Core Staple_97 |
| ATTCCTGATTAGAGAGATAAATTACGAGGCATAGTAA | Core Staple_98 |
| CAATATAATCCAAGTCAGAACCCTCGTTTACCAGACG | Core Staple_99 |
| TCTGAATAATGGACGGGAGTAGCGAGAGGCTTTTGCA | Core Staple_100 |
| TATCAAAATTAAGAATAACAGGGGGTAATAGTAAAAT | Core Staple_101 |
| TAAAGAAATTGTCAAAAATGTCCAATACTGCGGAATC | Core Staple_102 |
| CGTCAGATGAATCCAAATAGAATCCCCCTCAAATGCT | Core Staple_103 |
| TTACATCGGGACAAAATAAACGAGAATGACCATAAAT | Core Staple_104 |
| CTGATTGCTTTGCGTCTTTACCCTGACTATTATAGTC | Core Staple_105 |
| CGCGCAGAGGCTTTTATCCGCATCAAAAAGATTAAGA | Core Staple_106 |
| CCTGAGCAAAAGATTAGTTTCAAATATCGCGTTTTAA | Core Staple_107 |
| TCAAGAAAACACTTGCGGGAACCAGACCGGAAGCAAA | Core Staple_108 |
| ATTTCATTTGAGAACGCGATTAGAGAGTACCTTTAAT | Core Staple_109 |
| ACAGTACATAACAGATATAAGGTCATTTTTGCGGATG | Core Staple_110 |
| TAACCTTGCTTAATCATTACTGAATATAATGCTGTAG | Core Staple_111 |
| ATTAATTTTCCCAAGCAAGTATGCAACTAAAGTACGG | Core Staple_112 |
| TAGCGATAGCTTATTAAACTCCATATAACAGTTGATT | Core Staple_113 |
| GAGTCAATAGTGCTGTCTTAGTAGATTTAGTTTGACC | Core Staple_114 |
| GTCTGAGAGACTACGAGCAAAATGGTCAATAACCTGT | Core Staple_115 |
| TTAGGTTGGGTGAACAAGATTGGGGCGCGAGCTGAAA | Core Staple_116 |
| GCTGATGCAAAGCGCCTGTACTAATAGTAGTAGCATT | Core Staple_117 |
| ACGCGAGAAAAAATAAACATACAGGCAAGGCAAAGAA | Core Staple_118 |
| GTTAATTTCATAAGGTAAAATAAAGCCTCAGAGCATA | Core Staple_119 |
| GGTTTGAAATACAGTAATAACCAAAAACATTATGACC | Core Staple_120 |
| GGCGTTAAATACATGTAATGGAGAAGCCTTTATTTCA | Core Staple_121 |
| TAATTACTAGAATTGAGAATTTTAGAACCCTCATATA | Core Staple_122 |
| *CTCCTATCTCCAATCACTCCT*TCCAACGTGTTTAGTATTGATATAAGT | Handle staple for Cy3_1 |
| *CTCCTATCTCCAATCACTCCT*TTGGAACAAGAACCGCGTACCAGGCGG | Handle staple for Cy3_2 |
| *CTCCTATCTCCAATCACTCCT*AATAGCCCTCAGGGATGAAGGATTAGG | Handle staple for Cy3_3 |
| *CTCCTATCTCCAATCACTCCT*TCCGAAATCGTAACACAAAGTATTAAG | Handle staple for Cy3_4 |
| *CTCCTATCTCCAATCACTCCT*GCCCCAGCAACGCCTGCTATTTCGGAA | Handle staple for Cy3_5 |
| *CTCCTATCTCCAATCACTCCT*TGAGAGAGTTAGCGTAGTGCCCGTATA | Handle staple for Cy3_6 |
| *CTCCTATCTCCAATCACTCCT*GGGCAACAAGACGTTAGTTTTAACGGG | Handle staple for Cy3_7 |
| *CTCCTATCTCCAATCACTCCT*GCGCCAGGTGCTAAACTTTGATGATAC | Handle staple for Cy3_8 |
| *CTCCTATCTCCAATCACTCCT*GCCAACGCGAATAGAATTACCGTTCCA | Handle staple for Cy3_9 |
| *CTCCTATCTCCAATCACTCCT*GGAAACCTATAATTTTAAGCCAGAATG | Handle staple for Cy3_10 |
| *CTCCTATCTCCAATCACTCCT*AATTGCGTGGCTCCAATGATATTCACA | Handle staple for Cy3_11 |
| *CTCCTATCTCCAATCACTCCT*CCTGGGGTTCAGCTTGGGAGGTTGAGG | Handle staple for Cy3_12 |
| *CTCCTATCTCCAATCACTCCT*CACAACATTTGATACCCCACCACCAGA | Handle staple for Cy3_13 |
| *CTCCTATCTCCAATCACTCCT*TCCTGTGTCCATCGCCGAGCCACCACC | Handle staple for Cy3_14 |
| *CTCCTATCTCCAATCACTCCT*CGAGCTCGTGAGGCTTGAGCCGCCACC | Handle staple for Cy3_15 |
| *CTCCTATCTCCAATCACTCCT*GTGAGCCTGGATCGTCCCAGAGCCACC | Handle staple for Cy3_16 |
| *CTCCTATCTCCAATCACTCCT*TTTCTGCCGAACGAGGGCCATCTTTTC | Handle staple for Cy3_17 |
| *CTCCTATCTCCAATCACTCCT*ATGCGGCGGACTAAAGTTTTCGGTCAT | Handle staple for Cy3_18 |
| *CTCCTATCTCCAATCACTCCT*GCGCGCCTAACGGGTAGCGTCAGACTG | Handle staple for Cy3_19 |
| *CTCCTATCTCCAATCACTCCT*CCCCCTGCCCAACCTATCAGTAGCGAC | Handle staple for Cy3_20 |
| *CTCCTATCTCCAATCACTCCT*GATGCCGGAAAACACTCCAATGAAACC | Handle staple for Cy3_21 |
| *CTCCTATCTCCAATCACTCCT*CACTGGTGAAGCGCGACCATTACCATT | Handle staple for Cy3_22 |
| *CTCCTATCTCCAATCACTCCT*TAAAGGTTATCGCCTGGAATTAGAGCC | Handle staple for Cy3_23 |
| *CTCCTATCTCCAATCACTCCT*GCCGGGTCGCTCCATGTTATCACCGTC | Handle staple for Cy3_24 |
| *CTCCTATCTCCAATCACTCCT*CGCACTCAGGTCAATCTATTGACGGAA | Handle staple for Cy3_25 |
| *CTCCTATCTCCAATCACTCCT*TGTCCAGCGAAAGAGGCATTCAACCGA | Handle staple for Cy3_26 |
| *CTCCTATCTCCAATCACTCCT*GTGCCATCGCATAGGCGGTTTACCAGC | Handle staple for Cy3_27 |
| *CTCCTATCTCCAATCACTCCT*AACCGCAATGACAAGAATTTTGTCACA | Handle staple for Cy3_28 |
| *CTCCTATCTCCAATCACTCCT*AGAACGTCGTAACAAAAGAAACGCAAA | Handle staple for Cy3_29 |
| *CTCCTATCTCCAATCACTCCT*AGCACATCCCCTGACGAATACATACAT | Handle staple for Cy3_30 |
| *CTCCTATCTCCAATCACTCCT*TTTTTTCGGGGCTTGATTATTACGCAG | Handle staple for Cy3_31 |
| *CTCCTATCTCCAATCACTCCT*CGATGCTGTGTGAATTACCCAAAAGAA | Handle staple for Cy3_32 |
| *CTCCTATCTCCAATCACTCCT*CAGGCGGCCATTATACGAAACCGAGGA | Handle staple for Cy3_33 |
| *CTCCTATCTCCAATCACTCCT*ATCGACATTACGTTAAAAGCAGATAGC | Handle staple for Cy3_34 |
| *CTCCTATCTCCAATCACTCCT*CTCATTTGTTACAGGTTATCTTACCGA | Handle staple for Cy3_35 |
| *CTCCTATCTCCAATCACTCCT*GACGCAGAGAATACCAAAGAGCAAGAA | Handle staple for Cy3_36 |
| *CTCCTATCTCCAATCACTCCT*TCCGTGGTCCAAAAGGACCCACAAGAA | Handle staple for Cy3_37 |
| *CTCCTATCTCCAATCACTCCT*TACCAGTCCTATCATAGGGTAATTGAG | Handle staple for Cy3_38 |
| *CTCCTATCTCCAATCACTCCT*CGGAAACAAACCAAAAAATTAACTGAA | Handle staple for Cy3_39 |
| *CTCCTATCTCCAATCACTCCT*GAGGTGGATTTGCCAGATAAAAACAGG | Handle staple for Cy3_40 |
| *CTCCTATCTCCAATCACTCCT*ACGTTGTATGGATAGCGAAAATAGCAG | Handle staple for Cy3_41 |
| *CTCCTATCTCCAATCACTCCT*TAAGTTGGTATTCATTAGAAACGATTT | Handle staple for Cy3_42 |
| *CTCCTATCTCCAATCACTCCT*CAGCTGGCTTCAGAAAACAGCCATATT | Handle staple for Cy3_43 |
| *CTCCTATCTCCAATCACTCCT*AGGGCGATAGGTCTTTCCAGAGCCTAA | Handle staple for Cy3_44 |
| *CTCCTATCTCCAATCACTCCT*GCCATTCGAGCGGATTTGAATCTTACC | Handle staple for Cy3_45 |
| *CTCCTATCTCCAATCACTCCT*GGCACCGCGAAAGACTGCTATTTTGCA | Handle staple for Cy3_46 |
| *CTCCTATCTCCAATCACTCCT*GGCCTCAGTCAAAGCGAGGTTTTGAAG | Handle staple for Cy3_47 |
| *CTCCTATCTCCAATCACTCCT*GCATCTGCGGTCAGGAGGCGTTTTAGC | Handle staple for Cy3_48 |
| *CTCCTATCTCCAATCACTCCT*GTCACGTTTTGATAAGGAAGGCTTATC | Handle staple for Cy3_49 |
| *CTCCTATCTCCAATCACTCCT*GGGAACAACTTAATTGCCGCGCCCAAT | Handle staple for Cy3_50 |
| *CTCCTATCTCCAATCACTCCT*ATGTGAGCGTTTTAAACCGTTTTTATT | Handle staple for Cy3_51 |
| *CTCCTATCTCCAATCACTCCT*TGGCCTTCAGTTTCATCAAGTACCGCA | Handle staple for Cy3_52 |
| *CTCCTATCTCCAATCACTCCT*ACCAATAGTGCGAACGTCCTTATCATT | Handle staple for Cy3_53 |
| *CTCCTATCTCCAATCACTCCT*GCATTAAACATTTCGCTGTAGAAACCA | Handle staple for Cy3_54 |
| *CTCCTATCTCCAATCACTCCT*TTAAATTGATTTTCATAAAATAATATC | Handle staple for Cy3_55 |
| *CTCCTATCTCCAATCACTCCT*GCCCCAAATCAATTCTTTATCAACAAT | Handle staple for Cy3_56 |
| *CTCCTATCTCCAATCACTCCT*TCAATCATATAAATCAACATGTTCAGC | Handle staple for Cy3_57 |
| *CTCCTATCTCCAATCACTCCT*AGAATCGAATTAAGCAGTAATTCTGTC | Handle staple for Cy3_58 |
| *CTCCTATCTCCAATCACTCCT*TCAGGTCATCGGTTGTAGAGAATATAA | Handle staple for Cy3_59 |
| *CTCCTATCTCCAATCACTCCT*AGGGTAGCCTTTTGCGTTAGGCAGAGG | Handle staple for Cy3_60 |
| *CTCCTATCTCCAATCACTCCT*TTCAACCGATAAAAATTCGCCATATTT | Handle staple for Cy3_61 |
| *CTCCTATCTCCAATCACTCCT*GGCCGGAGGCAATGCCGCCAACGCTCA | Handle staple for Cy3_62 |
| *CTCCTATCTCCAATCACTCCT*CTCAGGAGCAAAGGGCGGAGCCCCCGA | Handle staple for Cy3_63 |
| *CTCCTATCTCCAATCACTCCT*CCACCCTCAGAGTCCACCGGCGAACGT | Handle staple for Cy3_64 |
| *CTCCTATCTCCAATCACTCCT*CCTCATTTGAGATAGGAAAGCGAAAGG | Handle staple for Cy3_65 |
| *CTCCTATCTCCAATCACTCCT*CCATGTACCGGCAAAAGCAAGTGTAGC | Handle staple for Cy3_66 |
| *CTCCTATCTCCAATCACTCCT*CAAACTACAGGCGAAAACCACACCCGC | Handle staple for Cy3_67 |
| *CTCCTATCTCCAATCACTCCT*CCTCATAGTTGCAGCACAGGGCGCGTA | Handle staple for Cy3_68 |
| *CTCCTATCTCCAATCACTCCT*GTCTTTCCGCTGATTGCACGTATAACG | Handle staple for Cy3_69 |
| *CTCCTATCTCCAATCACTCCT*TGGGATTTGTGGTTTTAGAGCGGGAGC | Handle staple for Cy3_70 |
| *CTCCTATCTCCAATCACTCCT*CGGAGTGAGCGGGGAGAGGGATTTTAG | Handle staple for Cy3_71 |
| *CTCCTATCTCCAATCACTCCT*TGCGAATAGTCGTGCCATCCTGAGAAG | Handle staple for Cy3_72 |
| *CTCCTATCTCCAATCACTCCT*AAAAAAAATGCGCTCAGCCACCGAGTA | Handle staple for Cy3_73 |
| *CTCCTATCTCCAATCACTCCT*TCGGTTTAGCCTAATGCAAATTAACCG | Handle staple for Cy3_74 |
| *CTCCTATCTCCAATCACTCCT*TAAACAGCACGAGCCGATTAGTAATAA | Handle staple for Cy3_75 |
| *CTCCTATCTCCAATCACTCCT*GACAACAAGAAATTGTAGAACTCAAAC | Handle staple for Cy3_76 |
| *CTCCTATCTCCAATCACTCCT*TCGGTCGCAATTCGTAATCCAGAACAA | Handle staple for Cy3_77 |
| *CTCCTATCTCCAATCACTCCT*CTTTTGCGCCTCACAGAACAGGAAAAA | Handle staple for Cy3_78 |
| *CTCCTATCTCCAATCACTCCT*CAGCATCGAGCACGCGATTTTGACGCT | Handle staple for Cy3_79 |
| *CTCCTATCTCCAATCACTCCT*GCTTTGAGGGCCGTTTATTTACATTGG | Handle staple for Cy3_80 |
| *CTCCTATCTCCAATCACTCCT*TTCCATTAGTGCACTCACCAGTAATAA | Handle staple for Cy3_81 |
| *CTCCTATCTCCAATCACTCCT*CGAAGGCAATCAGACGAGAGATAGAAC | Handle staple for Cy3_82 |
| *CTCCTATCTCCAATCACTCCT*AATACACTGTTACCTGAAGAATACGTG | Handle staple for Cy3_83 |
| *CTCCTATCTCCAATCACTCCT*ATTATACCTGTTCAGCATGGCTATTAG | Handle staple for Cy3_84 |
| *CTCCTATCTCCAATCACTCCT*TTTGTATCTCTTTGCTTAGCCCTAAAA | Handle staple for Cy3_85 |
| *CTCCTATCTCCAATCACTCCT*CGCGACCTACTGTTGCGAACGAACCAC | Handle staple for Cy3_86 |
| *CTCCTATCTCCAATCACTCCT*GCGCAGACATCCGCCGGGTGAGGCGGT | Handle staple for Cy3_87 |
| *CTCCTATCTCCAATCACTCCT*CCAACTTTATCAGCGGAACAGTGCCAC | Handle staple for Cy3_88 |
| *CTCCTATCTCCAATCACTCCT*GACCAGGCCCACGCAATGAAAAATCTA | Handle staple for Cy3_89 |
| *CTCCTATCTCCAATCACTCCT*AGTAATCTGAATGCCACTCAAATATCA | Handle staple for Cy3_90 |
| *CTCCTATCTCCAATCACTCCT*AAATCAACAGCGTGGTGTCAGTTGGCA | Handle staple for Cy3_91 |
| *CTCCTATCTCCAATCACTCCT*AAGGCTTGCTCATAACTTGAGGAAGGT | Handle staple for Cy3_92 |
| *CTCCTATCTCCAATCACTCCT*AGTAAATTTCTCGTCGAGCACTAACAA | Handle staple for Cy3_93 |
| *CTCCTATCTCCAATCACTCCT*TTAATCATATTGCCGTAATAGATAATA | Handle staple for Cy3_94 |
| *CTCCTATCTCCAATCACTCCT*AACTGGCTCTTTAGTGATTAGACTTTA | Handle staple for Cy3_95 |
| *CTCCTATCTCCAATCACTCCT*GAAAAATCAAAAAAATTATTAAATCCT | Handle staple for Cy3_96 |
| *CTCCTATCTCCAATCACTCCT*CAACATTACCGCCAGCTTTAAAAGTTT | Handle staple for Cy3_97 |
| *CTCCTATCTCCAATCACTCCT*AGATTTAGAACAGCGGCGGAACAAAGA | Handle staple for Cy3_98 |
| *CTCCTATCTCCAATCACTCCT*ACATAACGGAAGGGATATTATCATCAT | Handle staple for Cy3_99 |
| *CTCCTATCTCCAATCACTCCT*GAGCAACACCGGAATTTGGCAATTCAT | Handle staple for Cy3_100 |
| *CTCCTATCTCCAATCACTCCT*ACGATAAAATCGGCGATGGATTATACT | Handle staple for Cy3_101 |
| *CTCCTATCTCCAATCACTCCT*AAAGAAGTGCCGCCACAGAACCTACCA | Handle staple for Cy3_102 |
| *CTCCTATCTCCAATCACTCCT*GTTTAGACAAACGACGTAAAACAGAAA | Handle staple for Cy3_103 |
| *CTCCTATCTCCAATCACTCCT*GTCATAAAGTAACGCCTTCAGGTTTAA | Handle staple for Cy3_104 |
| *CTCCTATCTCCAATCACTCCT*TTAAACAGGAAAGGGGAACAGTACCTT | Handle staple for Cy3_105 |
| *CTCCTATCTCCAATCACTCCT*CAAAAATCCGGTGCGGAACGGATTCGC | Handle staple for Cy3_106 |
| *CTCCTATCTCCAATCACTCCT*AGAAGCAACCATTCAGAGTTACAAAAT | Handle staple for Cy3_107 |
| *CTCCTATCTCCAATCACTCCT*GGAAGCCCTTCTGGTGCATTTCAATTA | Handle staple for Cy3_108 |
| *CTCCTATCTCCAATCACTCCT*TTCGAGCTGAAGATCGTGAAACAAACA | Handle staple for Cy3_109 |
| *CTCCTATCTCCAATCACTCCT*CTCCAACACAGTTTGATACATTTAACA | Handle staple for Cy3_110 |
| *CTCCTATCTCCAATCACTCCT*TGCTCCTTGGTGTAGATTTTAATGGAA | Handle staple for Cy3_111 |
| *CTCCTATCTCCAATCACTCCT*GCTTAGAGACGGCGGAATGTGAGTGAA | Handle staple for Cy3_112 |
| *CTCCTATCTCCAATCACTCCT*CTCAACATGAGTAACACGTCGCTATTA | Handle staple for Cy3_113 |
| *CTCCTATCTCCAATCACTCCT*TGTCTGGACTGTAGCCCCTTGAAAACA | Handle staple for Cy3_114 |
| *CTCCTATCTCCAATCACTCCT*CCCAATTCGAACGCCAGACGCTGAGAA | Handle staple for Cy3_115 |
| *CTCCTATCTCCAATCACTCCT*ATTAGATATTTTTGTTCAAAATCATAG | Handle staple for Cy3_116 |
| *CTCCTATCTCCAATCACTCCT*TTAGCTATTAAACGTTTAACCTCCGGC | Handle staple for Cy3_117 |
| *CTCCTATCTCCAATCACTCCT*AGGTGGCAAACAGGAATATATGTAAAT | Handle staple for Cy3_118 |
| *CTCCTATCTCCAATCACTCCT*AACATCCAATGTACCCCAAGACAAAGA | Handle staple for Cy3_119 |
| *CTCCTATCTCCAATCACTCCT*TTAGCAAATGAACGGTAATATATTTTA | Handle staple for Cy3_120 |
| *CTCCTATCTCCAATCACTCCT*AAGCTAAATTGCCTGACTAAATTTAAT | Handle staple for Cy3_121 |
| *CTCCTATCTCCAATCACTCCT*CTGTAATATATTTTTGGTGATAAATAA | Handle staple for Cy3_122 |
| *CTCCTATCTCCAATCACTCCT*ACGCAAGGTTCTAGCTCACCGGAATCA | Handle staple for Cy3_123 |
| *CTCCTATCTCCAATCACTCCT*TTTTAAATACAGTCAAGTTTAGTATCA | Handle staple for Cy3_124 |
