# Supplementary material for: Stall force measurement of the kinesin-3 motor KIF1A using a programmable DNA origami nanospring
Source: eLife. 2026 Mar 25;14:RP108477. doi: 10.7554/eLife.108477 (PMC13016605; doi:10.7554/eLife.108477)
Supplement: Supplementary file 4. [file elife-108477-supp4.docx]

| **Sequence (5’ to 3’)** | **Name** |
| --- | --- |
| Cy3/AGGAGTGATTGGAGATAGGAG | Cy3-labeled antihandle |
