# Supplementary material for: Stall force measurement of the kinesin-3 motor KIF1A using a programmable DNA origami nanospring
Source: eLife. 2026 Mar 25;14:RP108477. doi: 10.7554/eLife.108477 (PMC13016605; doi:10.7554/eLife.108477)
Supplement: Supplementary file 5. [file elife-108477-supp5.docx]

| **Sequence (5’ to 3’)** | **Name** |
| --- | --- |
| AACGATCTAAAGTAAAGCCGGATTAAAGAACGTGGAC | Core Staple_01 |
| AGCATTCCACAGAAGAAAGCGAGTGTTGTTCCAGTTT | Core Staple_02 |
| GTTTCGTCACCAGGCAAGTGTATAAATCAAAAGAATA | Core Staple_03 |
| GCCCAATAGGAACCACACCCGTGATGGTGGTTCCGAA | Core Staple_04 |
| AGAGCCACCACCCGCGCGTACGCTGGTTTGCCCCAGC | Core Staple_05 |
| TCAGAACCGCCACTAACGTGCTGGCCCTGAGAGAGTT | Core Staple_06 |
| CACCGTACTCAGGAGCTAAACAGACGGGCAACAGCTG | Core Staple_07 |
| GTTGATATAAGTAGACAGGAAGGGCGCCAGGGTGGTT | Core Staple_08 |
| TCAGTACCAGGCGGTTTTTATGCCAACGCGCGGGGAG | Core Staple_09 |
| CAAGAGAAGGATTGAGTCTGTAAACCTGTCGTGCCAG | Core Staple_10 |
| AAACATGAAAGTAGCAATACTGCGTTGCGCTCACTGC | Core Staple_11 |
| GCCCCCTGCCTATTGCCTGAGGTGCCTAATGAGTGAG | Core Staple_12 |
| CTTGAGTAACAGTTGCTGGTAACGAGCCGGAAGCATA | Core Staple_13 |
| GTACTGGTAATAAGCCATTGCAATTGTTATCCGCTCA | Core Staple_14 |
| GCGTCATACATGGACCTACATCGTAATCATGGTCATA | Core Staple_15 |
| AAGCGCAGTCTCTGATTATTTAGTTGAGGATCCCCGG | Core Staple_16 |
| AACAAATAAATCCCGACCAGTTGCCTGTTCTTCGCGT | Core Staple_17 |
| GGCAGGTCAGACGAGAGATAGACGGTCATACCGGGGG | Core Staple_18 |
| CAGAGCCGCCGCCGAATACGTGTGCTGCGGCCAGAAT | Core Staple_19 |
| ACCACCCTCAGAGCTATTAGTCGCAGTGTCACTGCGC | Core Staple_20 |
| CCGCCACCCTCAGTAAAACATGGTGCCGGTGCCCCCT | Core Staple_21 |
| CAGAGCCACCACCCACCAGCACGGCATCAGATGCCGG | Core Staple_22 |
| TGCCATCTTTTCATCAGTATTCCCTTACACTGGTGTG | Core Staple_23 |
| GCATTTTCGGTCACTGAGAGCTGGGTAAAGGTTTCTT | Core Staple_24 |
| CTTTAGCGTCAGACATCACCTGAGCCGGGTCACTGTT | Core Staple_25 |
| ACCGTAATCAGTATCAATCAACGCACTCAATCCGCCG | Core Staple_26 |
| GAAACGTCACCAAAGTTGAAATCCAGCATCAGCGGGG | Core Staple_27 |
| CACCAGTAGCACCTATCTTTACATCCCACGCAACCAG | Core Staple_28 |
| TTGAGCCATTTGGGAGCCGTCAAGAATGCCAACGGCA | Core Staple_29 |
| TTCATTAAAGGTGAGAAGTATAGCGTGGTGCTGGTCT | Core Staple_30 |
| GAGGGAGGGAAGGCTCGTATTCATAACGGAACGTGCC | Core Staple_31 |
| GCCAAAGACAAAAATTTTAAAGTCGCTGGCAGCCTCC | Core Staple_32 |
| CAATCAATAGAAACGGAACAAGTTCCGGCAAACGCGG | Core Staple_33 |
| CAAAGACACCACGTATCATCAATGAAGGGTAAAGTTA | Core Staple_34 |
| ATACATAAAGGTGAATTCATCCGTAAAAAAAGCCGCA | Core Staple_35 |
| TTACGCAGTATGTATACTTCTGGGCGGTTGTGTACAT | Core Staple_36 |
| CCCAAAAGAACTGCCATATCACTTAAATTTCTGCTCA | Core Staple_37 |
| GGAAACCGAGGAAATAAAGAACGGAAAAAGAGACGCA | Core Staple_38 |
| AGTAAGCAGATAGGTCAGATGAGACTTTCTCCGTGGT | Core Staple_39 |
| ATAGCTATCTTACCATCGGGACATGTTTACCAGTCCC | Core Staple_40 |
| CAATAATAAGAGCTGCTTTGATCACCGGAAACAATCG | Core Staple_41 |
| AGAGAGATAACCCAGGCGAATCAGAGGTGGAGCCGCC | Core Staple_42 |
| ACAAAGTCAGAGGAAGAAGATACGTTGTAAAACGACG | Core Staple_43 |
| CATTAGACGGGAGAAATTAATAGTTGGGTAACGCCAG | Core Staple_44 |
| TACAGAGAGAATATACCTTTTTGGCGAAAGGGGGATG | Core Staple_45 |
| TGTTTAACGTCAAATATATGTATCGGTGCGGGCCTCT | Core Staple_46 |
| ATTTATCCCAATCATCGTCGCCCATTCAGGCTGCGCA | Core Staple_47 |
| AATTTGCCAGTTACCTTGAAACTGGTGCCGGAAACCA | Core Staple_48 |
| TACCAACGCTAACCGCTGAGAATCGCACTCCAGCCAG | Core Staple_49 |
| TTTGCACCCAGCTATCATAGGGAGGGGACGACGACAG | Core Staple_50 |
| TTTTGAAGCCTTACCGGCTTATGGGCGCATCGTAACC | Core Staple_51 |
| GCGTTTTAGCGAAAATGCTGAGACCGTAATGGGATAG | Core Staple_52 |
| AGAAGGCTTATCCAACGCGAGGTCGGATTCTCCGTGG | Core Staple_53 |
| TTACCGCGCCCAATTAATTTCCATCAACATTAAATGT | Core Staple_54 |
| GCAAGCCGTTTTTTTGAAATAAATTCGCGTCTGGCCT | Core Staple_55 |
| ATTAAACCAAGTATAAATAAGATTTTTTAACCAATAG | Core Staple_56 |
| GGCTGTCTTTCCTTAGAAAAAAAATTCGCATTAAATT | Core Staple_57 |
| ATTTACGAGCATGTACAAATTATATTTAAATTGTAAA | Core Staple_58 |
| GTCCTGAACAAGAAACAGTAGAAGCCCCAAAAACAGG | Core Staple_59 |
| GCAGAACGCGCCTTAACAACGTCAATCATATGTACCC | Core Staple_60 |
| GACGACGACAATAATTTTCGAAATCGATGAACGGTAA | Core Staple_61 |
| GAGCTTGACGGGGTTTGTCGTCAACAGTTTCAGCGGA | Core Staple_62 |
| AGAAAGGAAGGGACAGCCCTCACAACTAAAGGAATTG | Core Staple_63 |
| CGCTAGGGCGCTGTACAAACTCACGTTGAAAATCTCC | Core Staple_64 |
| TGCGCGTAACCACCCATGTACAAGGAGCCTTTAATTG | Core Staple_65 |
| GCGCCGCTACAGGTCATTTTCTGCTTTCGAGGTGAAT | Core Staple_66 |
| TGACGAGCACGTACCTCAGAATACCGATAGTTGCGCC | Core Staple_67 |
| GAATCAGAGCGGGAGGTTTAGATCGCCCACGCATAAC | Core Staple_68 |
| TAAAGGGATTTTATAGCCCGGTGAGGCTTGCAGGGAG | Core Staple_69 |
| AATCCTGAGAAGTGATAAGTGCGGGATCGTCACCCTC | Core Staple_70 |
| CCACCGAGTAAAAAGGATTAGATCGGAACGAGGGTAG | Core Staple_71 |
| ATTAACCGTTGTATTAAGAGGTTTGAGGACTAAAGAC | Core Staple_72 |
| TAATAACATCACTTTCGGAACTTCCATTAAACGGGTA | Core Staple_73 |
| AAACTATCGGCCTGCCCGTATTACGAAGGCACCAACC | Core Staple_74 |
| AATATTACCGCCAGTTTTAACAAAGAATACACTAAAA | Core Staple_75 |
| CGCTCATGGAAATCTTTTGATCCAGCGATTATACCAA | Core Staple_76 |
| ATCGTCTGAAATGGAATTTACAACGGAGATTTGTATC | Core Staple_77 |
| TTCACCAGTCACATCATTAAATGTCGAAATCCGCGAC | Core Staple_78 |
| CATTCTGGCCAACATTGGCCTAGCCGGAACGAGGCGC | Core Staple_79 |
| CCTGAAAGCGTAAAGCATTGAGGGAACCGAACTGACC | Core Staple_80 |
| TATTTTTGAATGGCCGCCACCAGATGAACGGTGTACA | Core Staple_81 |
| GAACTGATAGCCCAACCGCCATGGCTGACCTTCATCA | Core Staple_82 |
| ATACCGAACGAACGGAACCGCGAACCGGATATTCATT | Core Staple_83 |
| AGAGGTGAGGCGGTAATCAAACAAAGCTGCTCATTCA | Core Staple_84 |
| CAACAGTGCCACGTAGCCCCCCTGACGAGAAACACCA | Core Staple_85 |
| GAAAAATCTAAAGCTGTAGCGGGGCTTGAGATGGTTT | Core Staple_86 |
| AAATATCAAACCCGCGACAGAATTGTGAATTACCTTA | Core Staple_87 |
| TTGGCAAATCAACTGAAACCAGGCTCATTATACCAGT | Core Staple_88 |
| AGGTTATCTAAAAATTACCATAAAATCTACGTTAATA | Core Staple_89 |
| AACTAATAGATTAGAATTAGACAACATTATTACAGGT | Core Staple_90 |
| CATTTGAGGATTTAATTATCATGAGATTTAGGAATAC | Core Staple_91 |
| AACAATTCGACAATAAATATTAGATACATAACGCCAA | Core Staple_92 |
| CCGAACGTTATTAGGGCGACATAGTAAGAGCAACACT | Core Staple_93 |
| CATTATCATTTTGATTCATATACCAGACGACGATAAA | Core Staple_94 |
| GAAGGAGCGGAATGAATAAGTGGCTTTTGCAAAAGAA | Core Staple_95 |
| ATCAGATGATGGCGCAACATATAATAGTAAAATGTTT | Core Staple_96 |
| GATTGTTTGGATTTAGCAAACAATACTGCGGAATCGT | Core Staple_97 |
| GGGTTAGAACCTAGCATGATTATCCCCCTCAAATGCT | Core Staple_98 |
| ACGTAAAACAGAAACGCAATAACGAGAATGACCATAA | Core Staple_99 |
| TTTCAGGTTTAACCCGAACAATTACCCTGACTATTAT | Core Staple_100 |
| ACAGTACCTTTTACGAAGCCCGATTGCATCAAAAAGA | Core Staple_101 |
| GGATTCGCCTGATAAGAAACAAAGACTTCAAATATCG | Core Staple_102 |
| CAAAATCGCGCAGACAAGAATTCAAAGCGAACCAGAC | Core Staple_103 |
| ATTACCTGAGCAAGTAATTGACAGGTCAGGATTAGAG | Core Staple_104 |
| CATCAAGAAAACAAATTAACTCCTTTTGATAAGAGGT | Core Staple_105 |
| ATTTCATTTGAATACATAAAATTAGAGCTTAATTGCT | Core Staple_106 |
| AGTACATAAATCAAAATGAAACTCAACATGTTTTAAA | Core Staple_107 |
| CTTGCTTCTGTAACAAATAAGGGTGTCTGGAAGTTTC | Core Staple_108 |
| TTTCCCTTAGAATCAAAATAAGATTCCCAATTCTGCG | Core Staple_109 |
| GCTTAGATTAAGAGAGCGTCTTTGACCATTAGATACA | Core Staple_110 |
| TGAATTTATCAAAACAATTTTTAACCTGTTTAGCTAT | Core Staple_111 |
| ACCTTTTTAACCTAATCAAGACGAGCTGAAAAGGTGG | Core Staple_112 |
| ATAACTATATGTACCTCCCGAAGTAGTAGCATTAACA | Core Staple_113 |
| TCGCAAGACAAAGGGTATTCTGGCAAGGCAAAGAATT | Core Staple_114 |
| AAATATATTTTAGTAGCAAGCAAAGCCTCAGAGCATA | Core Staple_115 |
| TAAATTTAATGGTATTTTCATACCAAAAACATTATGA | Core Staple_116 |
| ATAAATAAGGCGTCCGCACTCCGGGAGAAGCCTTTAT | Core Staple_117 |
| GAATCATAATTACTATCATTCAAATTTTTAGAACCCT | Core Staple_118 |
| ATCATATGCGTTATAGAAACCAATGCCTGAGTAATGT | Core Staple_119 |
| AAAGCCAACGCTCAAAATAATAAGGGTGAGAAAGGCC | Core Staple_120 |
| GAATCGCCATATTGTTTATCACCATCAATATGATATT | Core Staple_121 |
| TTTAGGCAGAGGCAACAACATTAAATTAATGCCGGAG | Core Staple_122 |
| GAAACAGCTTCAGAAAATAACGGAATA | 398_501nm_Cy3nega_1 |
| GAAGGGATTCAGGTCTAGTTACCAGAA | 398_501nm_Cy3nega_2 |
| GGAATTTGGCAAAGCGTTTTTAAGAAA | 398_501nm_Cy3nega_3 |
| ACGGGAACTTCGAGCTTGAGTTAAGCC | 398_501nm_Cy3nega_4 |
| GCCAGTGCAACTCCAAGCGCTAATATC | 398_501nm_Cy3nega_5 |
| GGTTTTCCTAATTGCTGAACACCCTGA | 398_501nm_Cy3nega_6 |
| TGCTGCAACGGATGGCACAGGGAAGCG | 398_501nm_Cy3nega_7 |
| TCGCTATTTGCTGTAGATAGCAGCCTT | 398_501nm_Cy3nega_8 |
| ACTGTTGGTAAAGTACAAACGATTTTT | 398_501nm_Cy3nega_9 |
| ATCAAAAAAGCTCTCAATTGCGTAGAT | 398_501nm_Cy3nega_10 |
| TTAAGAGGTACAGCGCGAAACAATAAC | 398_501nm_Cy3nega_11 |
| CGTTTTAAGGATAACCATACCAAGTTA | 398_501nm_Cy3nega_12 |
| CGGAAGCACAAGCTTTTATTCATTTCA | 398_501nm_Cy3nega_13 |
| AGTACCTTCAGTCACGGATGAAACAAA | 398_501nm_Cy3nega_14 |
| CATTTTTGGGCGATTATACATTTAACA | 398_501nm_Cy3nega_15 |
| GAATATAAACGCCAGCTTAATGGAAAC | 398_501nm_Cy3nega_16 |
| TATGCAACGAAGGGCGGAGTGAATAAC | 398_501nm_Cy3nega_17 |
| GGCAAAGCTAACAGTTACAGCCATATT | 501_599nm_Cy3nega_1 |
| CTTTCCGGGATTTAGTTTCCAGAGCCT | 501_599nm_Cy3nega_2 |
| TATCGGCCATGGTCAAATCCTGAATCT | 501_599nm_Cy3nega_3 |
| GTGCATCTTTGGGGCGTTAGTTGCTAT | 501_599nm_Cy3nega_4 |
| GTCACGTTCTACTAATCTTGCGGGAGG | 501_599nm_Cy3nega_5 |
| GAACAAACATCATACAAAGAACGCGAG | 501_599nm_Cy3nega_6 |
| GAGCGAGTTAAGCAATAAATCAGATAT | 501_599nm_Cy3nega_7 |
| TCCTGTAGTCGGTTGTCGTAGGAATCA | 501_599nm_Cy3nega_8 |
| GAACGCCATACTTTTGATCGAGAACAA | 501_599nm_Cy3nega_9 |
| ATTCCATAGCCATTCGTATTAATTAAT | 501_599nm_Cy3nega_10 |
| AACGAGTACACCGCTTACATAGCGATA | 501_599nm_Cy3nega_11 |
| TTTCGCAATCAGGAAGAGAGTCAATAG | 501_599nm_Cy3nega_12 |
| ATTTTCATGCCAGTTTTCTGAGAGACT | 501_599nm_Cy3nega_13 |
| CATCAATTGGTGTAGAGGTTGGGTTAT | 501_599nm_Cy3nega_14 |
| TCCAATAAGGCGGATTTGCAAATCCAA | 501_599nm_Cy3nega_15 |
| AGCAAAATAACAACCCAAAACTTTTTC | 501_599nm_Cy3nega_16 |
| AAGCTAAACCAGCTTTATCTTCTGACC | 501_599nm_Cy3nega_17 |
| CCCTGTAATCAAAAATCCGACCGTGTG | 501_599nm_Cy3nega_18 |
| TTTGTTAAAAGGATAACAAGAACGGGT | 599_658nm_Cy3nega_1 |
| CGTTAATATTAAATGCAATCAATAATC | 599_658nm_Cy3nega_2 |
| AAGATTGTAGATTCAAATCCCATCCTA | 599_658nm_Cy3nega_3 |
| CGGTTGATTCAAATCAACAATAGATAA | 599_658nm_Cy3nega_4 |
| TCGTAAAACTAGCTGAGTTCAGCTAAT | 599_658nm_Cy3nega_5 |
| TTCAACGCATCAGCTCAATAAACACCG | 599_658nm_Cy3nega_6 |
| CATATATTTTTTGTTAGCCTGTTTAGT | 599_658nm_Cy3nega_7 |
| GTAGGTAAATAAGCAACTTACCAGTAT | 599_658nm_Cy3nega_8 |
| GGAGACAGAATCAGAAGGCTTAATTGA | 599_658nm_Cy3nega_9 |
| AGGGTAGCAACAAGAGGCCAGTAATAA | 599_658nm_Cy3nega_10 |
