# Supplementary material for: Stall force measurement of the kinesin-3 motor KIF1A using a programmable DNA origami nanospring
Source: eLife. 2026 Mar 25;14:RP108477. doi: 10.7554/eLife.108477 (PMC13016605; doi:10.7554/eLife.108477)
Supplement: Supplementary file 6. — Sequences in italics indicate the handle site. [file elife-108477-supp6.docx]

Handle staples for biotin

| **Sequence (5’ to 3’)** | **Name** |
| --- | --- |
| GGAACAAGAGAAAGGAATAGTTAGCGT | Handle for biotin #1 |
| *CTCTCCTCTCCACCATATCCA* |  |
| GTTACCTGCTTGACAACTCCCTCAGAG | Handle for biotin #2 |
| *CTCTCCTCTCCACCATATCCA* |  |
| GCGAAACGAAGCCCGAATGAAATAGCA | Handle for biotin #3 |
| *CTCTCCTCTCCACCATATCCA* |  |
| CTGGAGCATATTTTTGAATTCTGTCCA | Handle for biotin #4 |
| *CTCTCCTCTCCACCATATCCA* |  |
| TTTGCTAACAAAGGGCCCCCCGATTTA | Handle for biotin #5 |
| *CTCTCCTCTCCACCATATCCA* |  |
| GACCAGGCCGATCCAGCTTTAATGCGC | Handle for biotin #6 |
| *CTCTCCTCTCCACCATATCCA* |  |
| AGTCAGAATGAGAGATAATATACAGTA | Handle for biotin #7 |
| *CTCTCCTCTCCACCATATCCA* |  |
| CAACCGTTCTAGCATGCCAACATGTAA | Handle for biotin #8 |
| *CTCTCCTCTCCACCATATCCA* |  |

Handle staples for Cy3 fluorophore in a 398-nm long DNA calibration rod.

| **Sequence (5’ to 3’)** | **Name** |
| --- | --- |
| *CTCCTATCTCCAATCACTCCT*TCCAACGTACAACTTTCTTTCCAGACG | Handle staple for Cy3_398nm rod_1 |
| *CTCCTATCTCCAATCACTCCT*GCCCGAGAAATTTTTTACAACGCCTGT | Handle staple for Cy3_398nm rod_2 |
| *CTCCTATCTCCAATCACTCCT*ATCGGCAAGGCTCCAACGTAACACTGA | Handle staple for Cy3_398nm rod_3 |
| *CTCCTATCTCCAATCACTCCT*AGGCGAAATATCAGCTAGGGATAGCAA | Handle staple for Cy3_398nm rod_4 |
| *CTCCTATCTCCAATCACTCCT*GCAGCAAGCAGCTTGACCGCCACCCTC | Handle staple for Cy3_398nm rod_5 |
| *CTCCTATCTCCAATCACTCCT*ATTGCCCTCAACAACCTACCGCCACCC | Handle staple for Cy3_398nm rod_6 |
| *CTCCTATCTCCAATCACTCCT*TTTCTTTTTCGGTCGCAATAGGTGTAT | Handle staple for Cy3_398nm rod_7 |
| *CTCCTATCTCCAATCACTCCT*AGGCGGTTCGCTTTTGCCGTCGAGAGG | Handle staple for Cy3_398nm rod_8 |
| *CTCCTATCTCCAATCACTCCT*CTGCATTAAAGACAGCCGGGGTTTTGC | Handle staple for Cy3_398nm rod_9 |
| *CTCCTATCTCCAATCACTCCT*CCGCTTTCACAGAGGCCTGAGACTCCT | Handle staple for Cy3_398nm rod_10 |
| *CTCCTATCTCCAATCACTCCT*CTAACTCAGAGGAAGTCTATTATTCTG | Handle staple for Cy3_398nm rod_11 |
| *CTCCTATCTCCAATCACTCCT*AAGTGTAAAATGCCACAAACAGTTAAT | Handle staple for Cy3_398nm rod_12 |
| *CTCCTATCTCCAATCACTCCT*CAATTCCAAAGAGGCAGGGGTCAGTGC | Handle staple for Cy3_398nm rod_13 |
| *CTCCTATCTCCAATCACTCCT*GCTGTTTCTTTGACCCGATACAGGAGT | Handle staple for Cy3_398nm rod_14 |
| *CTCCTATCTCCAATCACTCCT*GTACCGAGCAAAGTACCGTTCCAGTAA | Handle staple for Cy3_398nm rod_15 |
| *CTCCTATCTCCAATCACTCCT*CCGTGAGCATAAATTGGCCAGAATGGA | Handle staple for Cy3_398nm rod_16 |
| *CTCCTATCTCCAATCACTCCT*TTTCTGCCTGTTACTTTGATATTCACA | Handle staple for Cy3_398nm rod_17 |
| *CTCCTATCTCCAATCACTCCT*GCGGCGGGAATCATAACAGGAGGTTGA | Handle staple for Cy3_398nm rod_18 |
| *CTCCTATCTCCAATCACTCCT*GCCTGTGCAAGAGGACAGAACCACCAC | Handle staple for Cy3_398nm rod_19 |
| *CTCCTATCTCCAATCACTCCT*GCATCAGAGCATAGGCCCCTCAGAGCC | Handle staple for Cy3_398nm rod_20 |
| *CTCCTATCTCCAATCACTCCT*TTCAGCAACAACGTAAATCACCGGAAC | Handle staple for Cy3_398nm rod_21 |
| *CTCCTATCTCCAATCACTCCT*TGCTCGTCGGCTTGCCTTATTAGCGTT | Handle staple for Cy3_398nm rod_22 |
| *CTCCTATCTCCAATCACTCCT*GCCCTGCGAGTAAATTCGTTTTCATCG | Handle staple for Cy3_398nm rod_23 |
| *CTCCTATCTCCAATCACTCCT*GGCGCGGTCTTTAATCATCAAGTTTGC | Handle staple for Cy3_398nm rod_24 |
| *CTCCTATCTCCAATCACTCCT*TCATTGCATAAGAACTTCGATAGCAGC | Handle staple for Cy3_398nm rod_25 |
| *CTCCTATCTCCAATCACTCCT*CTTACGGCTGGGAAGATAGCAAGGCCG | Handle staple for Cy3_398nm rod_26 |
| *CTCCTATCTCCAATCACTCCT*GCACCGTCTAACGGAAGCCAGCAAAAT | Handle staple for Cy3_398nm rod_27 |
| *CTCCTATCTCCAATCACTCCT*GGTCAGCATCATCAGTCCGTCACCGAC | Handle staple for Cy3_398nm rod_28 |
| *CTCCTATCTCCAATCACTCCT*GGACTTGTACTAATGCGACGGAAATTA | Handle staple for Cy3_398nm rod_29 |
| *CTCCTATCTCCAATCACTCCT*GGCCAGAGACGAGGCATTCAACCGATT | Handle staple for Cy3_398nm rod_30 |
| *CTCCTATCTCCAATCACTCCT*TCCGTTTTCCTCGTTTGGTTTACCAGC | Handle staple for Cy3_398nm rod_31 |
| *CTCCTATCTCCAATCACTCCT*AACGATGCTAGCGAGATTATTTTGTCA | Handle staple for Cy3_398nm rod_32 |
| *CTCCTATCTCCAATCACTCCT*CAGGCGGCAGAGGGGGTAAAAGAAACG | Handle staple for Cy3_398nm rod_33 |
| *CTCCTATCTCCAATCACTCCT*CGACATAATAGCGTCCGTAGAAAATAC | Handle staple for Cy3_398nm rod_34 |
| *CTCCTATCTCCAATCACTCCT*TTTGCCGCTTCATTGAAAGACTCCTTA | Handle staple for Cy3_398nm rod_35 |
| *CTCCTATCTCCAATCACTCCT*GTGAGAATAGTCCACTCGAACGTGGCG | Handle staple for Cy3_398nm rod_36 |
| *CTCCTATCTCCAATCACTCCT*CGAATAATTAGGGTTGAAAGGAGCGGG | Handle staple for Cy3_398nm rod_37 |
| *CTCCTATCTCCAATCACTCCT*AAAAAAAAAATCCCTTAGCGGTCACGC | Handle staple for Cy3_398nm rod_38 |
| *CTCCTATCTCCAATCACTCCT*TATCGGTTATCCTGTTCCGCGCTTAAT | Handle staple for Cy3_398nm rod_39 |
| *CTCCTATCTCCAATCACTCCT*TTCTTAAACGGTCCACTATGGTTGCTT | Handle staple for Cy3_398nm rod_40 |
| *CTCCTATCTCCAATCACTCCT*GACAATGATCACCGCCTTTCCTCGTTA | Handle staple for Cy3_398nm rod_41 |
| *CTCCTATCTCCAATCACTCCT*CGATATATCACCAGTGAGGAGGCCGAT | Handle staple for Cy3_398nm rod_42 |
| *CTCCTATCTCCAATCACTCCT*TTAAAGGCTGCGTATTCGGTACGCCAG | Handle staple for Cy3_398nm rod_43 |
| *CTCCTATCTCCAATCACTCCT*AGCAGCGAATGAATCGAATCAGTGAGG | Handle staple for Cy3_398nm rod_44 |
| *CTCCTATCTCCAATCACTCCT*CAACGGCTCAGTCGGGCCATCACGCAA | Handle staple for Cy3_398nm rod_45 |
| *CTCCTATCTCCAATCACTCCT*TTTTTCATCATTAATTTCTTTGATTAG | Handle staple for Cy3_398nm rod_46 |
| *CTCCTATCTCCAATCACTCCT*AAATACGTAGCCTGGGTAGAAGAACTC | Handle staple for Cy3_398nm rod_47 |
| *CTCCTATCTCCAATCACTCCT*TAAAACGACACAACATATATCCAGAAC | Handle staple for Cy3_398nm rod_48 |
| *CTCCTATCTCCAATCACTCCT*CACTCATCCTGTGTGAAACAGGAAAAA | Handle staple for Cy3_398nm rod_49 |
| *CTCCTATCTCCAATCACTCCT*GCGCGAAACTCGAATTTTTGACGCTCA | Handle staple for Cy3_398nm rod_50 |
| *CTCCTATCTCCAATCACTCCT*ATCGCCTGCTCCTCACACATTGGCAGA | Handle staple for Cy3_398nm rod_51 |
| *CTCCTATCTCCAATCACTCCT*CTGCTCCAAGCACGCGAATAAAAGGGA | Handle staple for Cy3_398nm rod_52 |
| *CTCCTATCTCCAATCACTCCT*AGACGGTCCCGTTTTCAACCCTTCTGA | Handle staple for Cy3_398nm rod_53 |
| *CTCCTATCTCCAATCACTCCT*AACTTTGAACTCTGTGGGCACAGACAA | Handle staple for Cy3_398nm rod_54 |
| *CTCCTATCTCCAATCACTCCT*AGAGTAATCAGCCAGCCGCCATTAAAA | Handle staple for Cy3_398nm rod_55 |
| *CTCCTATCTCCAATCACTCCT*ACCCAAATATCGTTAAGAAGATAAAAC | Handle staple for Cy3_398nm rod_56 |
| *CTCCTATCTCCAATCACTCCT*GTGAATAAATAAACATAACACCGCCTG | Handle staple for Cy3_398nm rod_57 |
| *CTCCTATCTCCAATCACTCCT*GAACGAGTGCTGGTAACAGCAGCAAAT | Handle staple for Cy3_398nm rod_58 |
| *CTCCTATCTCCAATCACTCCT*AATTTCAATGCGGTATTGCTGAACCTC | Handle staple for Cy3_398nm rod_59 |
| *CTCCTATCTCCAATCACTCCT*TGCGATTTGGCGCTTTTATCTGGTCAG | Handle staple for Cy3_398nm rod_60 |
| *CTCCTATCTCCAATCACTCCT*CAGGACGTTGGAGGTGGGAATTGAGGA | Handle staple for Cy3_398nm rod_61 |
| *CTCCTATCTCCAATCACTCCT*AAACGAACGGTGGTGCGGAGCACTAAC | Handle staple for Cy3_398nm rod_62 |
| *CTCCTATCTCCAATCACTCCT*AGAAAGATGCAACCGCAATAGATAATA | Handle staple for Cy3_398nm rod_63 |
| *CTCCTATCTCCAATCACTCCT*CACATTCAAGAACGTCTAGACTTTACA | Handle staple for Cy3_398nm rod_64 |
| *CTCCTATCTCCAATCACTCCT*AAGGAATTCACATCCTAAATCCTTTGC | Handle staple for Cy3_398nm rod_65 |
| *CTCCTATCTCCAATCACTCCT*ATCATAACTTCGTCTCAGTTTGAGTAA | Handle staple for Cy3_398nm rod_66 |
| *CTCCTATCTCCAATCACTCCT*AACCAAAATGATTGCCAGAAACCACCA | Handle staple for Cy3_398nm rod_67 |
| *CTCCTATCTCCAATCACTCCT*GTTTTGCCCTTTAGTGTATTCCTGATT | Handle staple for Cy3_398nm rod_68 |
| *CTCCTATCTCCAATCACTCCT*AGACTGGAAAAAATCCAATATAATCCT | Handle staple for Cy3_398nm rod_69 |
| *CTCCTATCTCCAATCACTCCT*CATAAATACAGCAGTTGAATAATGGAA | Handle staple for Cy3_398nm rod_70 |
| *CTCCTATCTCCAATCACTCCT*TTAAACAGGGATCAAAAAATTATTTGC | Handle staple for Cy3_398nm rod_71 |

Handle staples for Cy3 fluorophore in a 501-nm long DNA calibration rod. (The following handles were added along with those for the 398-nm long DNA calibration rod)

| **Sequence (5’ to 3’)** | **Name** |
| --- | --- |
| *CTCCTATCTCCAATCACTCCT*GAAACAGCTTCAGAAAATAACGGAATA | Handle staple for Cy3_501nm rod_1 |
| *CTCCTATCTCCAATCACTCCT*GAAGGGATTCAGGTCTAGTTACCAGAA | Handle staple for Cy3_501nm rod_2 |
| *CTCCTATCTCCAATCACTCCT*GGAATTTGGCAAAGCGTTTTTAAGAAA | Handle staple for Cy3_501nm rod_3 |
| *CTCCTATCTCCAATCACTCCT*ACGGGAACTTCGAGCTTGAGTTAAGCC | Handle staple for Cy3_501nm rod_4 |
| *CTCCTATCTCCAATCACTCCT*GCCAGTGCAACTCCAAGCGCTAATATC | Handle staple for Cy3_501nm rod_5 |
| *CTCCTATCTCCAATCACTCCT*GGTTTTCCTAATTGCTGAACACCCTGA | Handle staple for Cy3_501nm rod_6 |
| *CTCCTATCTCCAATCACTCCT*TGCTGCAACGGATGGCACAGGGAAGCG | Handle staple for Cy3_501nm rod_7 |
| *CTCCTATCTCCAATCACTCCT*TCGCTATTTGCTGTAGATAGCAGCCTT | Handle staple for Cy3_501nm rod_8 |
| *CTCCTATCTCCAATCACTCCT*ACTGTTGGTAAAGTACAAACGATTTTT | Handle staple for Cy3_501nm rod_9 |
| *CTCCTATCTCCAATCACTCCT*ATCAAAAAAGCTCTCAATTGCGTAGAT | Handle staple for Cy3_501nm rod_10 |
| *CTCCTATCTCCAATCACTCCT*TTAAGAGGTACAGCGCGAAACAATAAC | Handle staple for Cy3_501nm rod_11 |
| *CTCCTATCTCCAATCACTCCT*CGTTTTAAGGATAACCATACCAAGTTA | Handle staple for Cy3_501nm rod_12 |
| *CTCCTATCTCCAATCACTCCT*CGGAAGCACAAGCTTTTATTCATTTCA | Handle staple for Cy3_501nm rod_13 |
| *CTCCTATCTCCAATCACTCCT*AGTACCTTCAGTCACGGATGAAACAAA | Handle staple for Cy3_501nm rod_14 |
| *CTCCTATCTCCAATCACTCCT*CATTTTTGGGCGATTATACATTTAACA | Handle staple for Cy3_501nm rod_15 |
| *CTCCTATCTCCAATCACTCCT*GAATATAAACGCCAGCTTAATGGAAAC | Handle staple for Cy3_501nm rod_16 |
| *CTCCTATCTCCAATCACTCCT*TATGCAACGAAGGGCGGAGTGAATAAC | Handle staple for Cy3_501nm rod_17 |

Handle staples for Cy3 fluorophore in 599 nm DNA calibration rod. (The following handles were added along with those for the 398-nm and 501-nm long DNA calibration rod)

| **Sequence (5’ to 3’)** | **Name** |
| --- | --- |
| *CTCCTATCTCCAATCACTCCT*GGCAAAGCTAACAGTTACAGCCATATT | Handle staple for Cy3_599nm rod_1 |
| *CTCCTATCTCCAATCACTCCT*CTTTCCGGGATTTAGTTTCCAGAGCCT | Handle staple for Cy3_599nm rod_2 |
| *CTCCTATCTCCAATCACTCCT*TATCGGCCATGGTCAAATCCTGAATCT | Handle staple for Cy3_599nm rod_3 |
| *CTCCTATCTCCAATCACTCCT*GTGCATCTTTGGGGCGTTAGTTGCTAT | Handle staple for Cy3_599nm rod_4 |
| *CTCCTATCTCCAATCACTCCT*GTCACGTTCTACTAATCTTGCGGGAGG | Handle staple for Cy3_599nm rod_5 |
| *CTCCTATCTCCAATCACTCCT*GAACAAACATCATACAAAGAACGCGAG | Handle staple for Cy3_599nm rod_6 |
| *CTCCTATCTCCAATCACTCCT*GAGCGAGTTAAGCAATAAATCAGATAT | Handle staple for Cy3_599nm rod_7 |
| *CTCCTATCTCCAATCACTCCT*TCCTGTAGTCGGTTGTCGTAGGAATCA | Handle staple for Cy3_599nm rod_8 |
| *CTCCTATCTCCAATCACTCCT*GAACGCCATACTTTTGATCGAGAACAA | Handle staple for Cy3_599nm rod_9 |
| *CTCCTATCTCCAATCACTCCT*ATTCCATAGCCATTCGTATTAATTAAT | Handle staple for Cy3_599nm rod_10 |
| *CTCCTATCTCCAATCACTCCT*AACGAGTACACCGCTTACATAGCGATA | Handle staple for Cy3_599nm rod_11 |
| *CTCCTATCTCCAATCACTCCT*TTTCGCAATCAGGAAGAGAGTCAATAG | Handle staple for Cy3_599nm rod_12 |
| *CTCCTATCTCCAATCACTCCT*ATTTTCATGCCAGTTTTCTGAGAGACT | Handle staple for Cy3_599nm rod_13 |
| *CTCCTATCTCCAATCACTCCT*CATCAATTGGTGTAGAGGTTGGGTTAT | Handle staple for Cy3_599nm rod_14 |
| *CTCCTATCTCCAATCACTCCT*TCCAATAAGGCGGATTTGCAAATCCAA | Handle staple for Cy3_599nm rod_15 |
| *CTCCTATCTCCAATCACTCCT*AGCAAAATAACAACCCAAAACTTTTTC | Handle staple for Cy3_599nm rod_16 |
| *CTCCTATCTCCAATCACTCCT*AAGCTAAACCAGCTTTATCTTCTGACC | Handle staple for Cy3_599nm rod_17 |
| *CTCCTATCTCCAATCACTCCT*CCCTGTAATCAAAAATCCGACCGTGTG | Handle staple for Cy3_599nm rod_18 |

Handle staples for Cy3 fluorophore in 658 nm DNA calibration rod. (The following handles were added along with those for the 398-nm, 501-nm, and 599-nm long DNA calibration rod)

| **Sequence (5’ to 3’)** | **Name** |
| --- | --- |
| *CTCCTATCTCCAATCACTCCT*TTTGTTAAAAGGATAACAAGAACGGGT | Handle staple for Cy3_658nm rod_1 |
| *CTCCTATCTCCAATCACTCCT*CGTTAATATTAAATGCAATCAATAATC | Handle staple for Cy3_658nm rod_2 |
| *CTCCTATCTCCAATCACTCCT*AAGATTGTAGATTCAAATCCCATCCTA | Handle staple for Cy3_658nm rod_3 |
| *CTCCTATCTCCAATCACTCCT*CGGTTGATTCAAATCAACAATAGATAA | Handle staple for Cy3_658nm rod_4 |
| *CTCCTATCTCCAATCACTCCT*TCGTAAAACTAGCTGAGTTCAGCTAAT | Handle staple for Cy3_658nm rod_5 |
| *CTCCTATCTCCAATCACTCCT*TTCAACGCATCAGCTCAATAAACACCG | Handle staple for Cy3_658nm rod_6 |
| *CTCCTATCTCCAATCACTCCT*CATATATTTTTTGTTAGCCTGTTTAGT | Handle staple for Cy3_658nm rod_7 |
| *CTCCTATCTCCAATCACTCCT*GTAGGTAAATAAGCAACTTACCAGTAT | Handle staple for Cy3_658nm rod_8 |
| *CTCCTATCTCCAATCACTCCT*GGAGACAGAATCAGAAGGCTTAATTGA | Handle staple for Cy3_658nm rod_9 |
| *CTCCTATCTCCAATCACTCCT*AGGGTAGCAACAAGAGGCCAGTAATAA | Handle staple for Cy3_658nm rod_10 |
